# Pathogen-induced alterations in fine-scale movement behaviour predict impaired reproductive success

**DOI:** 10.1098/rspb.2025.0238

**Published:** 2025-04-09

**Authors:** Marius Grabow, Conny Landgraf, Juergen Niedballa, Carolin Scholz, Jan Pufelski, Ran Nathan, Sivan Toledo, Florian Jeltsch, Niels Blaum, Viktoriia Radchuk, Ralph Tiedemann, Stephanie Kramer-Schadt

**Affiliations:** ^1^ Technische Universität Berlin, Berlin, Germany; ^2^ Leibniz Institute for Zoo and Wildlife Research, Berlin, Germany; ^3^ Universität Potsdam, Potsdam, Germany; ^4^ Evolution, Ecology and Behavior, Movement Ecology Laboratory, Jerusalem, Israel; ^5^ Tel Aviv University Blavatnik School of Computer Science, Tel Aviv, Israel; ^6^ Universität Potsdam, Potsdam, Brandenburg, Germany; ^7^ Plant Ecology and Nature Conservation, Universitat Potsdam, Potsdam, Germany

**Keywords:** movement ecology, animal movement, host–pathogen dynamics, parental care, reproduction, habitat selection

## Abstract

Pathogens play an important role in ecosystems and may impair fitness-enhancing activities such as foraging. However, the sublethal effects of pathogens on host movement behaviour and their subsequent impacts on reproductive success are poorly understood. In this study, we used high-resolution tracking to examine the movements of free-ranging European starlings (*Sturnus vulgaris*) associated with sublethal avian blood parasite infections. We found that naturally infected individuals displayed reduced foraging behaviour, remained closer to their breeding location, and selected lower-quality habitats. These patterns were associated with poorer body condition of adults and less favourable development for their offspring. These behavioural changes suggest physiological limitations imposed by infection, reducing parental care and reproductive output. Our results provide compelling evidence that pathogen-induced changes in fine-scale movement behaviour are linked to impaired reproductive success, further emphasizing the need for a movement ecology perspective in local host–pathogen dynamics.

## 1. Introduction

Pathogens play pivotal roles in ecosystems, shaping species interactions, regulating populations and driving biodiversity patterns [1–3]. By imposing energetic costs on hosts, pathogens can reduce host fitness [4]. For example, decreased host survival due to infection is commonly observed in some host–pathogen systems, e.g. in highly pathogenic avian influenza [5]. Likewise, other pathogens reduce reproduction, as observed in mealworms (*Tenebrio molitor*) infected with tapeworms [6], directly impairing host fitness. However, hosts often tolerate infection caused by less virulent pathogens [7]. Therefore, behavioural alterations, such as the parasite-induced behavioural changes observable in numerous taxa [8], can serve as early indicators of pathogen impacts [9,10] and offer a more sensitive measure of pathogen effects than demographic metrics—particularly in cases of sublethal infection where fitness consequences are not immediately apparent [8,11,12].

Movement, here defined as whole organism displacement, is central to almost all fitness-enhancing activities [13]. Thus, alterations in movement behaviour caused by pathogens can lead to relative fitness losses compared to non-infected individuals [14]. For example, reduced movement behaviour has been associated with poorer body conditions [15,16]. This, in turn, could either prompt increased foraging to counteract weakened body condition, as observed in Atlantic cod (*Gadus morhua*) [17], or limit movement due to energetic constraints [18], highlighting the complexity of these interrelated factors. Understanding these effects through the lens of movement behaviour could therefore provide a crucial mechanistic insight into how pathogens contribute to relative fitness declines [12].

In this context, behavioural changes can be classified into two distinct phenomena. If driven by the host’s immune response, behavioural alterations are considered adaptive and termed sickness behaviour [19]. Common sickness behaviours include lethargy, reduced food intake, and social withdrawal [20]; all of which conserve energy for the immune system, potentially decreasing fitness losses. In contrast, physiological limitations result from the direct impacts of pathogens such as reduced oxygen transport to muscles and organs, tissue damage, or disrupted metabolic processes, leading to reduced whole organism’s performance capacity [12]. In these cases, alterations in movement behaviour, like more resting, are rather by-products of infection and do not serve as an adaptive mechanism to conserve energy, further impairing hosts capacity to perform tasks such as hunting, mating or foraging [8,12,15]. During energetically costly life history stages, such as the breeding period, pathogen-induced behavioural responses may become particularly pronounced, because hosts must increase their foraging efforts to not only maintain their own energetic needs but also to provide adequate resources for their offspring. Impaired movement during these times can severely hamper parental investment, which in turn affects offspring survival or condition [16,17]. In this context, early life-history stages such as the nestling period are particularly critical [21] because impairments in the ontogeny—the development and growth of an organism—will affect the survival and development, subtly affecting fitness and population dynamics [18].

Previous studies have not yet established a direct link between pathogens, movement behaviour, and reproductive success for two main reasons. Firstly, while some studies acknowledge the influence of pathogens, many fail to account for the infection status of individuals in wild species, leaving researchers uncertain whether individuals are infected or not [22]. This is especially true for detecting subclinical (‘hidden’) infections, when animals appear asymptomatic or are able to veil behavioural, physiological or morphological traits to some extent (e.g. [23,24]). Likewise, relating infection status to movement behaviour proves challenging because severely infected individuals, who are expected to move less, are rarely captured or tagged [25,26]. Consequently, the effects on those hosts that are infected but still engage in ’daily life’ are often subtle and thus require methods that are able to detect such minimal differences in relocation data [27]. Secondly, assessing the direct link between movement and reproduction requires consideration of appropriate spatiotemporal scales. For example, it is well established that the home range is the appropriate scale to examine the links between foraging and reproductive output (e.g [28,29]), encapsulated in Burt’s [30] seminal definition: ‘the area traversed by the individual in [an individual’s] normal activities of food gathering, mating and caring for young’. Consequently, we would expect that any reduction of this home range should link directly to reproductive success. Yet, detecting such changes in individual movement tracks requires sufficiently detailed movement data at the spatiotemporal scales relevant to foraging activities during the breeding period, including fine-scale habitat selection. Only recent advancements in animal tracking technology, such as the established high-throughput reverse GPS system known as ATLAS (Advanced Tracking and Localization of Animals in real-life Systems [31]), allow for such detailed analyses [27]. In combination with collecting life-history data of individuals, we can now directly link individual movement tracks to fitness proxies, paving the road for combining movement ecology with disease ecology [14].

Avian blood parasites (haemosporidia) that cause avian malaria, together with related parasites, exemplify how physiological limitations are challenging hosts’ performance. These vector-transmitted parasites spend parts of their complex life cycle within the red blood cells of infected birds where they feed on haemoglobin, leading to the destruction of these cells [23]. While often sublethal, the parasite impairs the host’s oxygen transport system, introducing anaemia, and reducing the host’s overall ability to perform vital activities. This has been demonstrated in reduced parental care in red-winged blackbirds (*Agelaius phoeniceus*) [24], decreased flight reactions in siskins (*Carduelis spinus*) [32] and altered migration in great reed warblers (*Acrocephalus arundinaceus*) [33]. Recently, the effects of blood parasites on survival were also observed while studying local movements at high temporal resolution, suggesting that infection drove resource selection and reduced survival in two passerine species, barn swallows (*Hirundo rustica*) and house martins (*Delichon urbicum*) [34]. Yet, to date no study exists that shows the direct link between movement, pathogens and reproductive output. While Hicks *et al*. [35], studying European shags (*Phalacrocorax aristotelis*), provide an energy-based perspective on reduced foraging behaviour without considering reproductive success, and Schoepf *et al*. [24] link infection status to reproduction without addressing movement decisions, our study bridges these gaps. By integrating all three elements (pathogens, movement behaviour and reproduction), we aim to offer a comprehensive perspective on how these elements interact.

**Figure 1.**
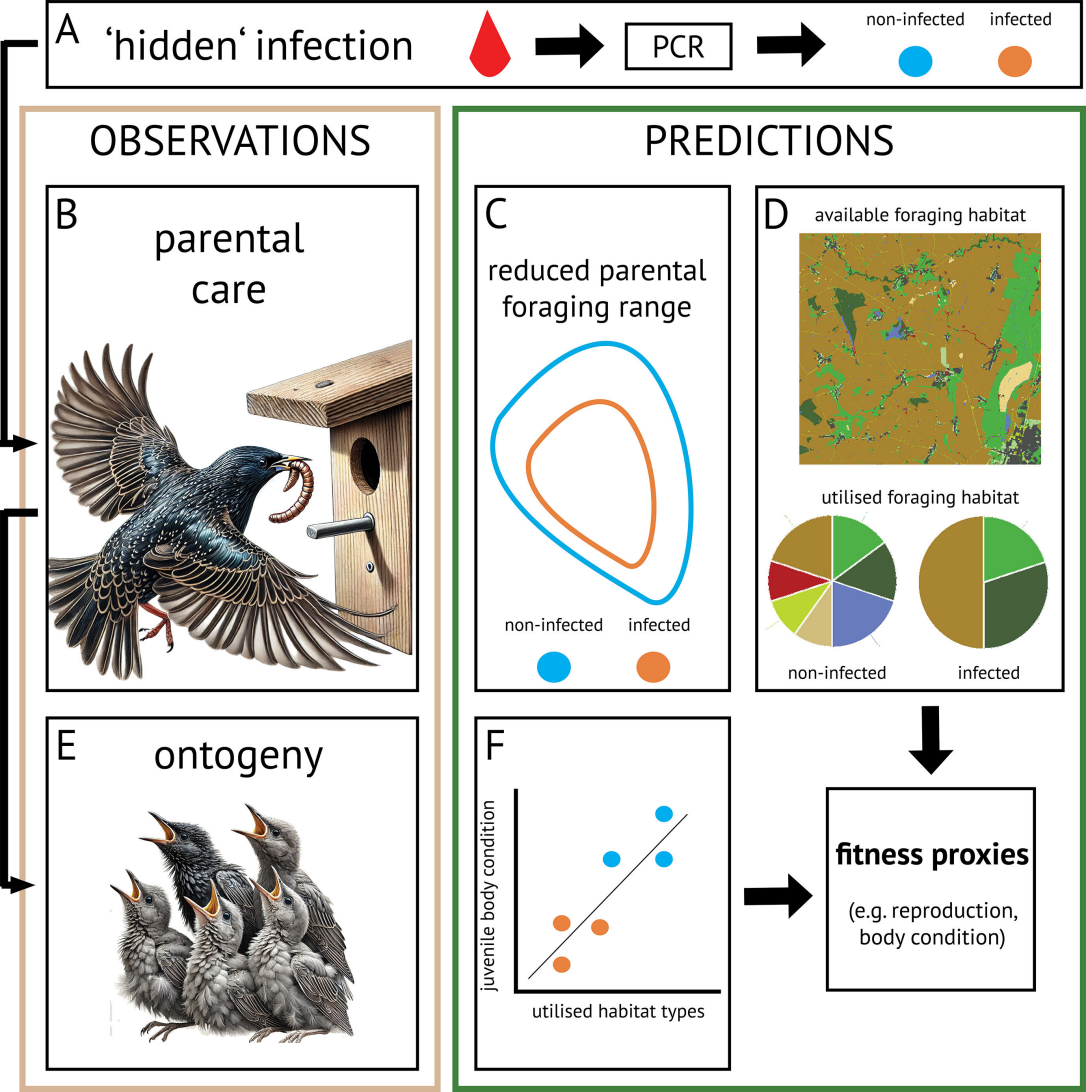
Overview over the hypothesis and predictions related to pathogen-induced alterations in foraging behaviour and subsequent consequences on fitness proxies, such as impaired reproduction or body conditions. (A) Infections that remain ‘hidden’ due to the absence of visible clinical signs require active testing for detection and classification of individuals’ infection status. In the case of avian blood parasites, this requires active collection of blood and analyses via PCR to obtain infection statuses. (B) By imposing costs on hosts, this infection can alter behaviour, including parental care movements. (C) Reduced movements may reduce parental foraging range sizes, and (D) restrict host’s foraging to specific habitat types, as resources are not distributed homogeneously. The map and pie chart are for illustrative purposes only, representing predicted foraging habitat utilization rather than actual distribution. For the distribution of habitat types, see figure 2C. (E) Juvenile development (ontogeny) is entirely dependent on the food provision of adults. (F) Therefore, reproductive success (measured as juvenile condition) is related to the foraging behaviour of adults. Eventually, the foraging behaviour of adults will determine fitness proxies, such as reproduction and regulation of body condition Starling illustrations created with the help of DALL·E 3.

Using high-resolution (0.125 Hz) movement data that allows uncovering subtle behavioural differences, we studied fine-scale habitat selection of European starlings (*Sturnus vulgaris*) in relation to their infection status and linked individual movements to their reproductive output. More specifically, we investigated the relationship between avian blood parasite infections and movement behaviour of adult nesting birds (figure 1A-B) in respect to their individual selection of habitat types for foraging. Our hypotheses and predictions were that (i) subclinically infected individuals, i.e. hosts that do not develop any clinical signs, would (ii) show reduced foraging behaviour and increased resting behaviour; (iii) infected individuals would have reduced foraging ranges (figure 1C), which in turn could be reflected by (iv) selecting different or less foraging habitats in comparison to non-infected individuals (figure 1D). Finally, we expected that (v) limitations in the foraging behaviour or the foraging ranges would correlate with reproductive performance (figure 1E) and predicted that parent birds that foraged less and chose less suitable foraging habitats would have (f) lower reproductive success, here measured as decreased juvenile body conditions, and decreased body conditions themselves, both being important proxies of fitness (figure 1F).

## 2. Methods

### (a) Study area and study species

**Figure 2.**
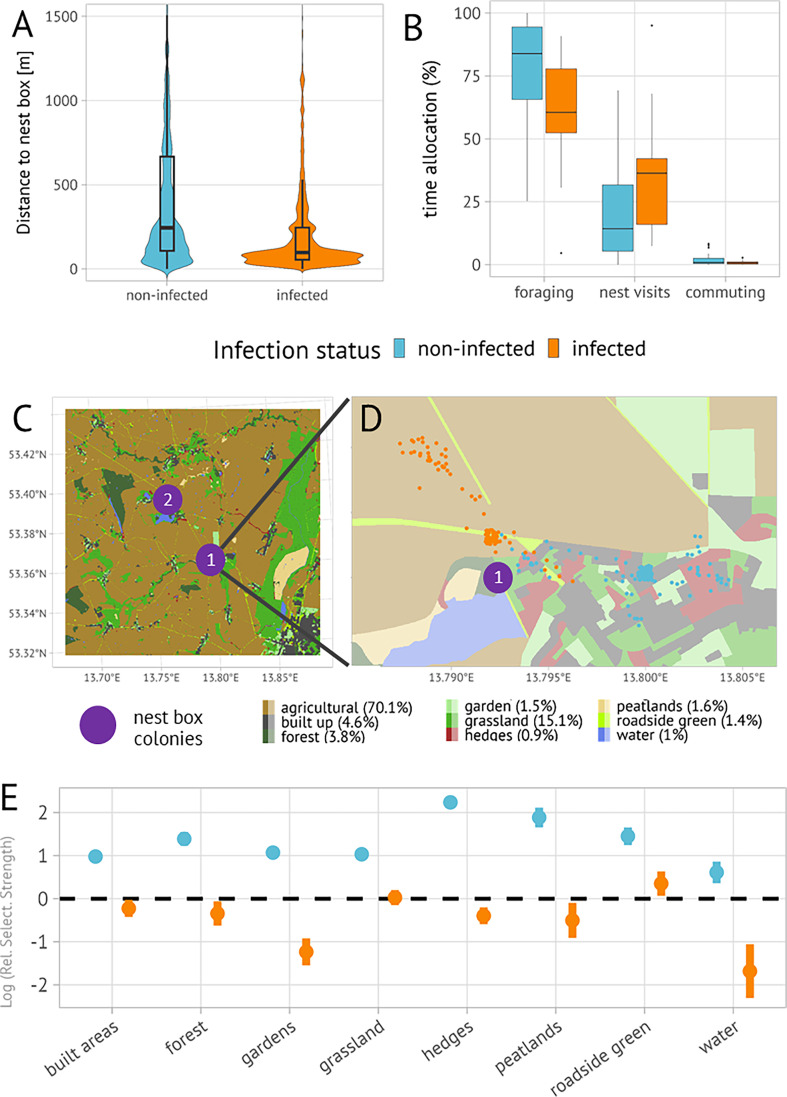
Differences in movement behaviour between non-infected and infected individuals. (A) Parental movement distances from nest box location. (B) Analyses of differences in behaviour states. (C) Map and distribution of habitat types, where non-agricultural habitats are not only patchily distributed but also scarce. (D) Exemplary high-resolution movement tracks (single feeding events between two nest-visits) of two different individuals, one being infected with a blood parasite (orange dots), the other one being non-infected (blue dots). While chosen opportunistically, these tracks exemplify observed movement differences. The movement track of the infected bird shows foraging (represented by point clouds) on roadside green and a second foraging stop on agricultural lands. In contrast, the non-infected individual selected for the urban area, foraging in gardens, along hedges, and grassland areas (E) Differences in habitat selection of non-infected and infected individuals, compared to foraging in agricultural areas (the reference category). Positive values indicate preferences for specific habitat types relative to agricultural areas, negative values indicate avoidance. Points represent mean values; error bars describe 95% CIs.

We conducted our study in northeast Germany (N 53.38°, E 13.75°), close to the city of Prenzlau, around 100 km north of Berlin. The area is predominantly used for agricultural purposes (70% of land use type) and encloses multiple small villages (5% of land use type) that are connected through smaller streets, typically surrounded by short strips of roadside green (1% of land use type). Grassland, including grazed habitat, contributes to 15% of all habitat types, private gardens and public green to 2% of all habitat types. Other habitat types are less clustered, rather patchily distributed and embedded into the agricultural matrix (figure 2C).

We conducted our study on a free-ranging population of European starlings (*S. vulgaris*), a native and common passerine bird that is highly abundant in our study area [36]. Nevertheless, starling population sizes are declining, especially in areas of agricultural intensification such as our study area [37]. Previous research linked these declines to the large losses of the preferred foraging habitat types of starlings—grazed habitat—where birds distinctly search for prey by probing, a technique that involves inserting the bill into the ground, which requires short vegetation cover [38–40] typically found in grazed habitats, mowed grasslands, private gardens or short roadside vegetation. Starlings are known hosts of multiple blood parasite genera such as *Plasmodium*, *Haemoproteus* and *Leucocytozoon* [41]; however, virulence is often expected to be low [42]. Avian blood parasites (haemosporidian parasites; families Plasmodiidae, Haemoproteidae and Leucocytozoidae) are transmitted via blood-sucking vectors, distributed globally and lead to various immune responses in hosts. Common vectors include mosquitos (Culicidae), biting midges (Ceratopogonidae) and black flies (Simuliidae) [43]. Previous research could link impairments in host health mainly to the acute phase of infection when consumption of haemoglobin is high in host cells, which may disrupt red blood cells and leads to anaemia [44].

### (b) Animal capturing and tracking study

By setting up a total of 35 nest boxes, we established two colonies (figure 2C). Both colony locations are similar regarding their surrounding habitat types, i.e. both are located close to small lakes on grassland that grows relatively high and thus cannot be used for foraging by starlings (figure 2C,D). Therefore, starlings typically forage at other habitat types (grassland, private gardens, agricultural land, or roadside vegetation) that are in close vicinity. Utilizing nest boxes equipped with remote closing mechanisms, we captured adults directly at their nest site, which allowed assessing their reproductive outputs with certainty (electronic supplementary material S1). We studied starling movement behaviour during their nest visits between 2021 and 2023, specifically when they were engaging in parental care activities such as brooding, feeding or nest cleaning activities. To reduce disturbances during the particularly sensitive early breeding stages, we captured adult birds when their juveniles were at least 5 days old.

Upon capture, we ringed each individual and took morphological measurements of adult birds, including tarsometatarsus length (mm; i.e. the measurement of the fused ankle-foot bone in birds, serving as an indicator of skeletal size), body weight (g), wing length (mm), 8th primary feather length (mm), tail length (mm), and tail fork width (mm). We estimated fat scores following Kaiser [45], ranging from 1 (minimal fat) to 8 (high-fat storage), and estimated muscle scores following Bairlain *et al*. [46] between 0 (muscle depressed) and 3 (full rounded muscle). For each individual, we calculated individual body condition as weight divided by tarsometatarsus length. We punctured the brachial vein of all captured individuals with a hollow needle, collected approx. 30 µl of blood, and stored them in stabilization buffer. We extracted DNA and used polymerase chain reaction (PCR; electronic supplementary material S2) for the identification of blood parasite genera [47]. Eventually, we pooled blood parasite genera to obtain a binary infection status for each individual (i.e. individuals are either non-infected or infected).

To assess the reproductive traits of adult starlings, we surveyed nest boxes closely from the first nest-building activities until fledging. Typically, starling nest-building activities begin in April, with nests being finalized within few days (2–3 days), incubation taking 11–13 days, and hatching occurring 16–20 days later [48]. We collected egg-laying dates and counted the number of eggs (clutch size), juveniles (number of hatched nestlings) and assessed the number of fledged juveniles (observation via regular nest box monitoring) if nest boxes were empty and we would not find signs of predation events. We took morphological measurements of juveniles when they were exactly 15 days old (i.e. 14 days after their individual hatching date); we ringed all juveniles, took individual weight and tarsometatarsus length and calculated body conditions (weight/tarsometatarsus).

We tracked adult starling movement behaviour using ATLAS [31]. We equipped adult starlings with 1.6 g ATLAS transmitters (tag-to-body weight ratio: 2.06% ± 0.01 (mean ± s.d.); range: 1.91%–2.34%) with a sampling interval of 0.125 Hz (i.e. one localization every 8 s), using a figure-eight harness that retained starling’s flight ability [49]. All spatial locations were calculated via ATLAS’s ‘robust algorithm’ [50]. We removed localizations with high uncertainty and removed all localizations collected during night, as our tracking system was partially operating utilizing solar energy and turned off during night to conserve energy for daytime operation, making these observations less accurate (electronic supplementary material S3). As we were interested in parental foraging movements, we removed all localizations after the juveniles reached an age of 15 days, when we measured morphological traits of the juveniles. For each localization, we calculated the Euclidean distance to the respective nest box. We defined the area within 150 m around the nest box as indicative of a ‘home visit’, typically representing parental care when adults feed their juveniles [48]. This radius was chosen because it is smaller than the typical foraging range if grassland is not in close vicinity [37,51], however, including potential resting occurring close to the nest box location (e.g. in the treetop). Similarly, we considered the interval between two ‘home visits’ as one foraging trip (figure 2D).

### (c) Statistical analysis

We ran all statistical analyses in R 4.3.3 [52]. For machine learning classifications of movement data (see 2.4.2), we utilized Keras 2.11.0 [53] and TensorFlow 2.11 [54].

#### (i) Movement behaviour: parental foraging ranges

For each individual, we estimated space to use during parental care movements by fitting continuous time movement models using the ‘ctmm’ package in R [55] and obtained 95% autocorrelated kernel density estimates (aKDE). We refer to this range distributions as ‘parental foraging ranges’. To address missing localizations caused by receiver-transmitter malfunctions, we simulated these based on the fitted ctmm for short-time lags, maintaining appropriate autocorrelation and localization error models. For larger time gaps (≥ 25 missing localizations; 224 s), which typically occur while individuals are foraging on the ground, we reconstructed paths manually by simulating localizations close to the last observation (electronic supplementary material S3).

#### (ii) Movement behaviour: behavioural classification of movement data

We used machine-learning to classify behavioural states from individual foraging trips. Analyses were focused on foraging trips above 3 minutes (i.e. 3198 trips; 1 343 000 localizations) to not include very short trips that are presumably not related to any parental foraging movements. We randomly selected 10% of the foraging trips (i.e. 319 trips; 129 000 localizations) as training data and used QGIS (version 3.22.6) to classify each localization based on the expected behaviour. During the breeding season, starling foraging behaviour is distinct and thus easy to identify: Birds leave the nest box, commute to a foraging site, and return to feed their nestlings. We distinguished three different behaviours (feeding of nestlings at their colony nest box, foraging, and commuting between foraging site and nest box), and one class for faulty localizations (e.g. if a tag localization produced high uncertainty). We evaluated regression techniques (k-nearest neighbour, KNN) and six different recurrent neural networks (RNNs) on the test data using a standardized training regime with 50 epochs, a batch size of 256 and a validation split of 20%. The models included a simple RNN, long short-term memory (LSTM), gated recurrent unit (GRU) and three variations of bidirectional LSTMs (electronic supplementary material S4). Each model was fitted with a categorical cross-entropy loss function, the root mean square propagation (RMSprop) optimizer with a learning rate of 0.0001. Early stopping was implemented to avoid overfitting and performance metrics for each model including loss and accuracy were recorded. We generated confusion matrices to assess the classification performance. Ultimately, one model (see §3c) was chosen that offered a balanced trade-off between accuracy and other performance metrics, using the loss function as a reference to identify behavioural states.

#### (iii) Movement behaviour: foraging habitat selection

We fitted habitat selection functions (HSFs) on the group level, comparing foraging habitat selection (i) between infected and non-infected individuals and (ii) for each individual separately. We focused habitat selection analyses on behaviour that was classified as ‘foraging’ via our machine learning classifications (see §2c(ii)). To reduce spatial autocorrelation, we resampled movement tracks to a 30 min resolution (electronic supplementary material S5). For each observed foraging location, we drew 20 random points (i.e. available) from the study area (figure 2C) and extracted the underlying habitat types using the ‘*amt’* package [56]. We used agricultural habitat as our intercept reference category, being the predominant habitat type and presumably unsuitable foraging habitat [38,40,57]. To correct for the imbalance in numbers between observed and random locations (1 : 20), we assigned weights (10 000 : 1) to each random location and included this bias correction in the likelihood function. We fitted a generalized linear model (GLM) for the group level (i) by comparing true with available steps, using an interaction term between infection status and habitat type. To obtain selection coefficients per individual (ii), we repeated model fitting for each individual using GLMs. We choose this two-step modelling because a unified model with random slopes and intercept per individual and habitat type showed convergence issues.

#### (iv) Predictors of juvenile and adult body condition

We performed a two-way ANOVA examining the effects of blood infection and sex on adult body condition. To assess if clutch sizes and number of nestlings would differ based on the infection status, we performed Poisson GLMs. To identify movement-related associations with juvenile and adult body condition, we fitted a multivariate Bayesian model using scaled juvenile and adult body conditions as response variables, with individual habitat selection coefficients as predictors (see §2d(iii)). Additionally, we tested additive effects of parental infection on juvenile body condition and of infection status on adult body condition. We scaled the response variable juvenile body condition, and adult body condition was scaled by sex to account for sexual dimorphism. We used uninformative priors for all variables. We fitted models via ‘*brms*’ [58] in R by running Markov chain Monte Carlo (MCMC) algorithms with four chains, each with 5000 iterations including a burn-in period of 1000 samples. We assessed chain convergence by measuring the Gelman–Rubin statistic for each chain and report estimates as mean and 89% credible intervals (CIs) as suggested by Kruschke [59].

## 3. Results

### (a) Capturing, tracking and infection status

We captured 29 adult starlings (male = 12; female = 17). The majority of individuals were from the main colony (*n* = 26), the other individuals (*n* = 3) were nesting in a second colony (figure 2C). The total blood parasite prevalence was 37.9% including all three parasite genera, of which *Haemoproteus* infection (31.04% of all individuals) was the most common. One individual had a co-infection with two *Haemoproteus* species and one individual was carrying a co-infection with *Plasmodium* and *Leucocytozoon*. We collected a total number of 2 120 000 localizations until juveniles reached an age of 15 days.

### (b) Parental foraging distances and ranges

Parental foraging distances from the nest box location were significantly smaller in infected individuals (Wilcoxon rank-sum test: W = 7.1663 × 10^11^, *p* < 0.001). Specifically, non-infected individuals mean distance from the nest box was 493 m ± 660 m (mean ± s.d.) while it was 219 m ± 328 m (mean ± s.d.) for infected individuals (figure 2A). The parental foraging ranges were around 35% smaller in infected individuals (mean_infected_: 1.23 km² [CI95: 0.4−2.8 km²]; mean_non-infected_: 1.68 km² [CI95: 0.4−4.9 km²]; *Χ*^2^-inverse Gaussian hierarchical model, *p* = 0.33) than in non-infected individuals. We found distinct coefficients of variation (CoVs), indicating small variation in parental foraging ranges for infected individuals (CoV: 1.72 [CI95: 0.65−2.82]), and large variation in the size of parental foraging ranges in non-infected individuals (CoV: 3.22 [CI95: 0.93−5.60]). Parents performed on average 1077 ± 1129 (mean ± s.d.) foraging trips during that period (see electronic supplementary material, video S1). We found neither statistically significant differences in the number of foraging trips between infected and non-infected individuals (Wilcoxon rank-sum test: W = 189, *p* = 0.132), nor in the cumulative distance of trips (Wilcoxon rank-sum test: W = 116, *p* = 0.717).

### (c) Behavioural classification and habitat selection

On average, non-infected individuals spent 76.9% ± 20.6% (mean ± s.d.) of the tracked period foraging, 20% ± 19.1% (mean ± s.d.) at nest locations, and 1.94% ± 2.45% (mean ± SD) commuting. Infected individuals foraged significantly less, 59.8% ± 25.0% (Wilcoxon rank-sum test: W = 204, *p* = 0.043; figure 2B), but had a non-significant tendency to spend more time at their nest location; 36.2% ± 26.6 (Wilcoxon rank-sum test: W = 77, *p* = 0.071; figure 2B). There was no difference in commuting behaviour between infected and non-infected individuals (Wilcoxon rank-sum test: W = 155, *p* = 0.430; figure 2B). Model selection revealed that LSTM was best in predicting behavioural states (electronic supplementary material S4). After training, the model achieved an accuracy of 90.68%, with a loss value of 0.2838, indicating a sufficient balance between accuracy and model generalization. The training process converged smoothly, with minimal variance between training epochs.

Our habitat selection model on the group level (i.e. differences between infected and non-infected individuals) revealed highly contrasting trends of habitat selection between infected and non-infected individuals for many habitat types (figure 2E). Specifically, compared to agricultural habitats (the reference category), non-infected individuals foraged significantly more (relative probability: mean: 78% ± 8%, range 65%–90%) in all other habitat types (figure 2E). In contrast, infected individuals foraged less often in other habitat types than agricultural land (relative probability: 33% ± 12%, range 16%–44%), with two exceptions: they showed a slight preference for roadside green (relative probability: 59% ± 53%), and exhibited no clear preference for or against grassland (figure 2E). Individual foraging habitat selection estimates were incorporated as predictors for juvenile and adult body conditions (see §3d).

### (d) Reproductive success

#### (i) Reproduction: number of offspring, juvenile body condition and parental provisioning

We collected 105 individual body measurements of juvenile starlings from different nest boxes (*n* = 22) when they were 15 days old. On average, nest boxes contained 4.77 ± 1.19 (mean ± s.d.) juveniles that fledged successfully. Juveniles weighed 63.5 g ± 10.7 g (mean ± s.d.) and had an average body condition of 2.2 g mm^–1^ ± 0.3 g mm^–1^ (mean ± s.d.). A two-way ANOVA revealed a significant effect of year (*F*_(2,101)_ = 10.98, *p* < 0.001), and a significant effect of parental infection status on juvenile body condition; *F*_(1,101)_ = 16.04, *p* < 0.001. Tukey post-hoc tests showed that juveniles had significantly lower body conditions in 2022 and 2023 compared to 2021 (mean = −0.25 and −0.33, respectively, both *p* < 0.001), but no significant difference between 2022 and 2023 (mean = −0.08, *p* = 0.45). Note that the means are unitless as body condition values were scaled prior to analyses (see §2). Moreover, juveniles with infected parents had significantly lower body conditions compared to those with non-infected parents (mean = −0.18, *p* < 0.001). The proportion of time spent in the behavioural state ‘foraging’ did not significantly influence juvenile body condition (β = −0.001, t(98) = −1.008, *p* = 0.316). We found neither significant differences in the clutch sizes between infected and non-infected individual (Poisson GLM: *β* = 0.962, s.e. = 0.17, *z* = −0.05, *p* = 0.962) nor in the number of juveniles (Poisson GLM: *β* = 0.07, s.e. = 0.27, *z* = −0.31, *p* = 0.755).

#### (ii) Reproductive output, body condition and habitat selection estimates

**Figure 3.**
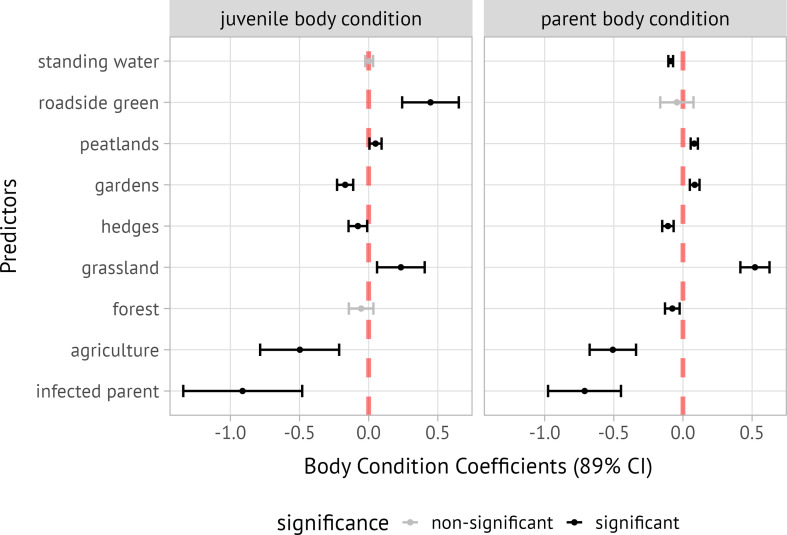
Coefficient plot showing predictors of juvenile and adult body condition related to adult foraging habitat selection and infection status. Points represent mean estimates; error bars indicate 89% credible intervals. Positive coefficients (e.g. grassland) indicate that stronger foraging habitat selection for the habitat type is associated with increased body condition in both juveniles and adults. Negative coefficients (e.g. agricultural land) reflect associations with decreased body conditions. The additive effects of parental infection status consistently correspond to reduced body condition in both juveniles and adults.

The relationship between blood infection and adult body condition was not statistically significant in general (*F*₁,₂₅ = 1.243, *p* = 0.2755), while sex had a significant effect on body condition, with males being larger (*F*₁,₂₅ = 4.495, *p* = 0.0441). However, adult infection status was associated with a significant decrease of both adult and juvenile body condition in the following models that incorporate individual movement decisions. In the Bayesian multivariate model, all MCMC chains converged, indicated by Rhat values of 1.00 for all parameters. The Bayesian R-squared value for the model was estimated at 0.40 (89% CI: 0.28, 0.50) for juvenile body condition and 0.69 (89% CI: 0.62, 0.74) for adult body condition. The strongest predictor of decreased body condition in both juveniles and adults was the presence of, or being, an infected parent (juveniles: −0.91, 89% CI: −1.35, −0.48; adults: −0.71, 89% CI: −0.97, −0.45). This trend was driven by a weak but statistically significant correlation between infection and foraging habitat selection for agricultural land (*Χ*^2^ (1) = 100.47, *p* = 0.001; Cramér’s *v* = 0.148). Among habitat types, the selection of agricultural habitat was the most important predictor of decreased body condition (juveniles: −0.50, 89% CI: −0.78, −0.21; adults: −0.51, 89% CI: −0.68, −0.33). In contrast, the selection of grassland was associated with increased body condition (juveniles: 0.23, 89% CI: 0.06, 0.41; adults: 0.52, 89% CI: 0.42, 0.63). For juveniles, parents selecting roadside green habitat also predicted increased body condition (0.45, 89% CI: 0.24, 0.65), while other habitat selection coefficients had smaller and sometimes contrasting effects (figure 3).

## 4. Discussion

In this study, we employed high-resolution tracking to investigate the movements of free-ranging European starlings, uncovering distinct behavioural variations in parental foraging linked to sublethal natural avian blood parasite infections. We found compelling evidence that pathogen-induced changes in fine-scale movement behaviour are significantly correlated with habitat selection of foraging adults during the breeding period. More importantly, these behavioural alterations were associated with negative juvenile development, specifically impaired morphological traits, and correlated with reduced adult body condition. While correlational, we establish a clear link between parasitic infection, movement behaviour, and reproductive success, demonstrating how subtle shifts in local movement may lead to fitness deficiencies. Our findings offer a potential explanation for how pathogens impose fitness costs through altered movement behaviour, and highlight the value of studying animal movement at finer spatiotemporal scales to detect even nuanced behavioural effects [27].

Our finding that infected individuals foraged closer to their nest boxes and spent less time foraging suggests reduced performance capacity—the ability to perform fitness-enhancing tasks [12]. This is further supported by the greater variation in parental care ranges among non-infected individuals, while infected individuals consistently showed smaller foraging ranges. This pattern indicates physiological restrictions limiting movement in infected birds. The increased nest-feeding behaviour in infected individuals, despite reduced foraging, may reflect physiological constraints commonly associated with acute infections, as observed in experimental studies [23,32,44]. Similar physiological limitations have been documented in free-ranging European shags (*Phalacrocorax aristotelis*) equipped with accelerometers, which revealed pathogen-induced reduction of daily energy budgets [35]. In European starlings, however, the physiological effects of infection remain unstudied. While our research provides a potential behavioural explanation, it focuses on external factors, leaving the underlying physiological mechanisms speculative. Nonetheless, modified movement behaviour due to infection is commonly observed in many host–pathogen systems (e.g. [8,25,60–62]) and was furthermore confirmed by a recent meta-analysis comparing non-infected and infected migratory animals [63]. While such studies, studying long-distance movements, provide valuable insights into pathogen transport over long distances and delayed migrations [33,64–66], they typically fall short of linking these behavioural changes to specific habitat utilization, let alone fitness components. Here, our research bridges a crucial gap by incorporating not only the differences in local habitat use between infected and non-infected individuals but incorporating potential fitness proxy feedbacks.

Our behavioural classifications revealed a significant decrease in time allocated to foraging behaviour and increased nest-visiting behaviour in infected individuals. Decreased foraging behaviour during infection might seem counterintuitive given the typically increased energetic demands caused by infection [67]. However, it may be explained by the need for increased resting, as observed in various passerine species [68], with the resting occurring spatially apart from the foraging location. In social foragers like starlings, which typically forage with conspecifics to avoid predators [69], another factor may be important. Starlings are highly mobile during foraging, moving frequently within and between patches, likely led by non-infected or stronger individuals. Weaker, infected birds, needing to keep up with conspecifics, may be forced to reduce foraging duration and remain closer to the nest to conserve energy. A similar trend has been observed in white storks (*Ciconia ciconia*), where young birds struggled to keep up with mixed flocks during migration, increasing their energetic demands, which negatively impacted juvenile survival [70]. Previous experimental work on starlings revealed such positive relationship between flock density and foraging duration [71]. This could explain the reduced time spent foraging by infected adults, the positive correlation between parental foraging range size and adult body condition, and the impaired body condition in their offspring if adults reduced the time spent foraging. However, due to the correlational nature of this study, we cannot entirely rule out the possibility that individuals who rest more, for unrelated reasons, are more prone to infection. This would suggest that we are measuring behavioural types rather than pathogen-induced effects [72]. While theoretically possible, this scenario is unlikely given evidence from experimental studies showing that increased resting behaviour is more commonly a response to infection [32,61,68], furthermore confirmed in a recent empirical study analysing local movement behaviour of wild swallow species [34]. In our study, however, this resting behaviour occurred almost exclusively at the individual’s nest box location. Since adults were sitting near the nest without providing food items (as observed opportunistically via camera traps and during occasional monitoring of nest boxes via binoculars), typically the most important form of parental care during the late breeding season [73], this behaviour should not be misinterpreted as enhanced parental care, especially given the observed decline in body condition of both adults and offspring.

Differences in resource utilization between infected and non-infected individuals are commonly observed in animals and are often driven by sickness behaviour. For example, sheep (*Ovis aries*) infected with gastrointestinal parasites modify their foraging behaviour to counteract the effects of infection [74]. In birds infected with avian blood parasites, migratory individuals have been shown to prioritize energy-rich nutrients to counterbalance increased metabolic demands [67]. On the other hand, behavioural changes can also stem from the infection’s direct physiological impact, such as reduced mobility or compromised energy efficiency [8]. For instance, reef fish (*Haemulon flavolineatum*) infected by *Anilocra haemuli* move less due to physiological costs of infection [75]. In our study, the differences in foraging habitat selection are more likely attributable to these physiological constraints, as infected individuals stayed closer to their colony with the vicinity offering only lower quality foraging habitats.

Our findings particularly emphasize the stark differences in space use between infected and non-infected individuals, especially regarding agricultural areas. These areas are widely known to have reduced food availability (e.g. [76]). While non-infected individuals largely avoided these lower-quality habitats, our results suggest that infected birds need to forage there, probably due to their inability to access more preferred habitat types. This trend should be particularly notable in starlings, which typically avoid intensified agricultural landscapes under normal conditions [38–40,51]. While reduced foraging ranges or altered behaviour are not disadvantageous *per se*—and may even conserve energy by limiting flight distances [77]—this is only beneficial if high-quality habitat or successful foraging opportunities are available. As a result, high-quality habitat may buffer the negative effects of parasitic infection, if infected individuals are still able to collect resources efficiently. In human-altered environments, nesting and foraging habitats are often spatially separated, forcing animals to forage farther from their nesting sites (e.g. [78]).

In this context, our study found a similarly reduced offspring body condition of infected individuals, which is associated with reduced foraging behaviour and the use of less energy-rich habitats compared to uninfected conspecifics. This suggests that offspring condition is linked to the feeding behaviour of adults [79,80]. This was also confirmed experimentally in starlings [29]. Consistent with the biology of starlings, stronger selection of grassland and roadside green—both characterized by short grass preferred for foraging—was associated with increased offspring body condition. Conversely, stronger selection for agricultural land, which has lower insect abundance [81], explained the poorer body condition of offspring. It is important to note that this study uses juvenile and adult body condition as a proxy for fitness. While tracking an individual’s lifetime reproductive success would be an ideal measurement of fitness, it is often unfeasible in long-lived species [82]. However, previous studies show that lower body weights are an early indicator of long-term fitness decline in multiple bird species, including starlings [83,84] (but see [85]). Additionally, interruptions in juvenile development may also signal challenges that impair songbird’s future reproductive success [21,86].

Given the correlational nature of our study, it is important to consider whether the observed differences in behaviour, resource selection, adult body condition and offspring quality could stem from factors other than infection, such as pre-existing fitness deficiencies or individual phenotypic traits. For instance, individuals that forage less—for unrelated reasons—may also be more prone to infection and select agricultural habitats more frequently. While we cannot fully reject this hypothesis due to the limitations inherent in correlational studies, there is evidence against it. Particularly, if fitness deficiencies were the primary cause for the observed pattern, we would also expect measurable signs of reduced fitness proxies, such as reduced body condition, in the infected individuals. However, we found no evidence that these traits were different in general, and were only different in models that included movement behaviour and body condition. Furthermore, behavioural alterations caused by infection are highly plausible, given that infection can impose physiological constraints [23], which should directly affect movement and foraging behaviour.

One potential alternative explanation for the observed relationship between adult movement behaviour and juvenile body condition is that juveniles were infected via their parents if there is a blood-sucking vector in the nest. While possible in theory, detectable infections typically do not occur within the prepatent period, up to three weeks [23]. Given that our study focused on juveniles at 15 days of age, it is highly unlikely that juvenile infection with the same parasite occurred during this period. These biological constraints allow us to exclude juvenile infection with avian blood parasites as a confounding factor, reinforcing the conclusion that adult movement behaviour directly influences juvenile body condition. However, reduced body condition in juveniles and adults may also result from co-infections with other pathogens beyond the scope of this study. Co-infections are common, particularly in individuals with suppressed immune systems [87,88], and additional parasites may further deplete host energy reserves or be directly transmitted to offspring, potentially producing the same patterns we observed. The list of potential co-infections is extensive, and screening for multiple infections is often constrained by limited samples and high costs. Therefore, future studies should, where ethically feasible, adopt experimental approaches to disentangle the effects of movement behaviour and pathogen interactions [25].

Another important caveat of this study is the use of a binary infection status (infected versus non-infected) rather than infection intensity. The nested PCR method we employed is highly sensitive, detecting even low parasite loads [47], which may include chronically infected individuals or those with reduced parasitaemia and minimal physiological effects. Binary classification has limitations and previous studies found them to be inconclusive, as low parasitaemia often results in only minor physiological consequences [44]. Data on parasitaemia in future studies could strengthen our conclusions by addressing the study’s correlative nature. For instance, a correlation between high parasitaemia and increased resting time in infected individuals would further support our findings and could corroborate the physiological consequences observed in previous studies [32,44]. On the other hand, even chronic avian malaria was shown to severely affect fitness [89], underscoring the importance of our study as a key step towards linking pathogen-induced changes in movement to reproductive traits. This warrants further investigation into these complex interactions and the effects of proximate, reactive traits like movement behaviour to ultimate consequences in fitness.

We are still far from accurately predicting future scenarios regarding pathogens’ shifting distributions and their effects on ecosystems [90]. However, growing evidence suggests that ecosystems subjected to multiple stressors [91], such as those facing the combined impacts of climate change and land use alterations, are becoming increasingly vulnerable to altered host–pathogen dynamics (e.g. [92,93]). Our research serves as an early warning of these potential effects, particularly if current land use changes and fragmentations continue, increasing the need to travel farther distances between nesting and foraging sites, potentially limiting hosts' ability to counteract infections. Though traditionally regarded as relatively benign, avian blood parasites are already linked to observable changes in behaviour, body condition and offspring quality. Our findings may serve as a blueprint for understanding the broader effects of pathogens, as similar effects may be observable in many host–pathogen systems where small changes may have profound effects on animal populations.

## Data Availability

The movement data used for this study is contained in a Movebank repository (Movebank ID: 3028843612). All computer code to reproduce the study is publicly available at [94]. Supplementary material is available online [95].
